# Adult retrospective report of child abuse and prospective indicators of childhood harm: a population birth cohort study

**DOI:** 10.1186/s12916-021-02164-5

**Published:** 2021-11-29

**Authors:** Snehal M. Pinto Pereira, Nina T. Rogers, Christine Power

**Affiliations:** 1 Division of Surgery & Interventional Science, University College London, London, UK; 2 UCL Great Ormond Street Institute of Child Health, University College London, London, UK

**Keywords:** Epidemiology, Child abuse, Longitudinal, Recall bias

## Abstract

**Background:**

We aim to determine whether adult retrospective report of child abuse is associated with greater risk of prospectively assessed harmful environments in childhood. We assessed possible recall basis by adult depression status.

**Methods:**

At 45 years, participants of the 1958 British birth cohort (*N* = 9308) reported a range of abuse types (by 16 years). Prospective data, ages 7–16 years, were obtained for impoverished upbringing, hazardous conditions, anti-social behaviours and 16 years poor parent-child relationships. We estimated associations between retrospective report of child abuse and prospectively measured harm using (i) odds ratios (*OR*s, 95% confidence intervals) and (ii) positive predictive values (PPVs). PPVs were calculated stratified by adult depression status.

**Results:**

Prevalence of retrospectively reported abuse ranged from 10.7% (psychological) to 1.60% (sexual) and 14.8% reported ≥ 1 type; prospectively recorded harm ranged from 10% (hazardous conditions/poor parent-child relationships) to 20% (anti-social behaviours). Adults retrospectively reporting abuse were more likely to have had harmful childhood environments: 52.4% had ≥ 1 indicator of harm (vs. 35.6% among others); *OR*_sex-adjusted_ for poor relationships with parents was 2.98 (2.50, 3.54). For retrospectively reported (vs. none) abuse, there was a trend of increasing relative risk ratio with number of harms, from 1.75 (1.50, 2.03) for 1 to 4.68 (3.39, 6.45) for 3/4 childhood harms. The PPV of ≥ 1 prospectively recorded harm did not differ between depressed (0.58 (0.52, 0.64)) and non-depressed (0.58 (0.55, 0.61)) groups.

**Conclusions:**

In a population cohort, adult retrospective report of child abuse was associated with several harms, prospectively measured from childhood to adolescence, providing support for the validity of retrospective report-based research. Findings suggest retrospectively reported child abuse is not biased by depression in adulthood.

**Supplementary Information:**

The online version contains supplementary material available at 10.1186/s12916-021-02164-5.

## Background

Abuse in childhood has been linked to several poor health and socioeconomic outcomes throughout the life-course, including poorer emotional [1] and physical [2, 3] health as well as inflammation [4] and pre-mature mortality [5, 6]. Additionally, abuse is associated with lower educational achievement and increased risk of financial and employment-related difficulties [7, 8]. Child abuse is not uncommon [9]: globally, approximately 36% of children are estimated to have been psychologically abused and 23% physically abused [10]. To date, although some child abuse research is based on prospective measures [3, 11], much of the research relies on retrospective report.

There are several potential shortcomings of retrospectively reported (i.e. recalled) abuse. In particular, recall may be imprecise on the temporal sequence of events, for chronicity and severity, and may also be vulnerable to bias, e.g. retrospectively reported abuse could be biased by current mental health [12, 13]. Indeed, ascertainment of child abuse for research purposes is not straightforward, with acknowledged limitations for all available methods [9]. Accordingly, whilst prospective abuse measurement may avoid recall bias, it is not without limitations [14]. Notably, prospective measures may miss a substantial number of cases that systematically differ from others [15], possibly capturing only the most severe cases of abuse (e.g. those that come to the attention of child protection services via educational, criminal-justice, social-services or healthcare personnel) [16]. The possibility that prospective identification underestimates the incidence of abuse in the general population is supported by a systematic review using international classifications of diseases, finding that abuse was underreported in health data systems [17]. Given that both prospective and retrospective measures of abuse are imperfect, any differences in agreement between these measures cannot simply be interpreted as poor validity of retrospective assessments [18].

For research purposes, retrospective measures have been important in establishing long-term health risks associated with abuse [1, 5, 19–21] and they are often the only practical option for case ascertainment. Thus, it is likely that retrospective reports of abuse will continue to be used. However, there is a need to gain further insights into the meaning of such retrospective reports. One potentially promising approach would be to compare adult retrospective reports of child abuse with prospectively recorded indicators of harm that are expected to be linked to abuse. Relevant proxy indicators are suggested by a broad literature linking abuse to specific features of the child’s environment, e.g. (i) impoverished upbringing (requiring welfare support or institutional care); (ii) hazardous conditions (indicated by injuries and hospitalisation); (iii) anti-social behaviours indicating distress and/or dysfunction and (iv) poor parent–child relationships. For example, the literature indicates that children exposed to severe maltreatment are likely to have extensive contact with children’s social care (and other specialist agencies) [22], including child protection services [23]. Also, child abuse has been linked to increased risk of hospitalisations [24], and injuries including bone fractures [25] and burns [26, 27] such that multiple injuries have been documented as important manifestations of child abuse [26]. Anti-social behaviours in childhood/adolescence may also be indicative: e.g. abuse has been associated with fighting and disobedience [28] and with an approximate doubling in the likelihood of criminal behaviour [29]. In addition, a meta-analysis suggested strong links of physical abuse with family conflict and low family cohesion [30].

Proxy indicators of abuse may be imperfect with respect to accurate identification of individuals’ cases of abuse. However, they may be informative in suggesting whether retrospectively reported abuse is linked to the harmful environments (mentioned above) that are expected to be related to abuse. Given that for pragmatic reasons, retrospectively reported abuse will continue to be used for research purposes, it is important to build understanding of how retrospective reports relate to conditions during which the abuse might have occurred. We do this by exploiting the wealth of prospectively collected data in the 1958 British birth cohort and examine whether adult retrospective report of child abuse is associated with greater risk of harmful environments prospectively assessed from childhood to adolescence. Specifically, in this general population, we examine the extent to which retrospectively reported child abuse is related to prospective measures of (i) impoverished upbringing, (ii) hazardous conditions, (iii) anti-social behaviours and (iv) poor parent-child relationships. A secondary aim is to assess the potential of recall basis linked to current mental health status in adulthood, to influence adult reports of abuse in childhood. Specifically, we examine whether associations between retrospective reports of abuse and prospective indicators of harm differed by adult mental health status.

## Methods

The 1958 British birth cohort includes over 17,000 participants enrolled at birth during 1 week in March 1958 in Scotland, Wales and England [31]. Information was collected at birth and follow-ups at ages 7 years, 11 years, 16 years, 23 years, 33 years, 42 years, 45 years, 50 years and 55 years. Immigrants to Britain born during the same week in 1958 were included into the survey at 7 years, 11 years and 16 years. At survey sweeps, data were collected from multiple sources, including participants, parents, teachers, health visitors and doctors. Participants gave informed consent at several of the surveys and ethical approval was given, including at 45 years, by the South East Multi-centre Research Ethics committee. The sample for analysis in the current study consists of those with information on adult retrospective reports (at 45 years) of different types of child abuse (*N* = 9308). Respondents at 45 years are broadly representative of the surviving cohort [32].

### Measures

#### Retrospective reports of child abuse

Physical, psychological, witnessing and sexual abuse (up to 16 years) by a parent/parent-figure was reported by participants at 45 years during a home interview with a trained nurse. Participants recorded information using a confidential computer-assisted data-entry questionnaire derived from the Personality and Total Health Through Life Project [33], originating from the Parental Bonding Instrument [34], British National Survey of Health and Development [35] and the US National Comorbidity Survey [36] (details in Table 1).

**Table.1:** Retrospectively reported* childhood abuse: definition of different types, representative variables and prevalence in the 1958 British birth cohort

Child abuse type	Definition^a^	1958 cohort variables	Prevalence *N* (%)
Physical abuse (0–16 years)	Intentional use of physical force or implements against a child that results in, or has the potential to result in, physical injury.	I was physically abused by a parent—punched, kicked or hit or beaten with an object or needed medical treatment	562 (6.04)
Psychological abuse^b^ (0–16 years)	Intentional behaviour that conveys to a child that s/he is worthless, flawed, unloved, unwanted, endangered or valued only in meeting another’s needs. *UK definition*^c^ *includes harmful (unintentional) parent-child interactions: ‘the persistent emotional maltreatment of a child such as to cause severe and persistent adverse effects on the child’s emotional development’*	- I was verbally abused by a parent (or parent-figure) - I suffered humiliation, ridicule, bullying or mental cruelty from a parent (or parent-figure) - Mother (or mother-figure) and father (or father-figure) were not at all affectionate	1000 (10.7)
Witnessing abuse (0–16 years)	Any incident of threatening behaviour, violence or abuse (psychological, physical, sexual, financial or emotional) between intimate partners or adult family members, irrespective of sex or sexuality.	I witnessed physical or sexual abuse of others in my family	559 (6.01)
Sexual abuse (0–16 years)	Any completed or attempted sexual act, sexual contact or non-contact sexual interaction with a child by a caregiver.	I was sexually abused by a parent (or parent-figure)	149 (1.60)
Any abuse (0–16 years)		Experiencing at least one of physical, psychological, witnessing or sexual abuse (as described above)	1381 (14.8)

Total sample: 9308.

^*^Reported at 45 years. Information was obtained via direct computer data entry. Participants were instructed: “The following are statements about your childhood. For each, please say whether the statement applies to you.” Response options were “Yes,” “No” or “Can’t say”

^a^[16]

^b^Defined as a report of at least one of the three listed variables

^c^Department for Education. Working together to safeguard children. Her Majesty’s Government, 2006

#### Prospective indicators of harmful environments in childhood and adolescence

Based on the literature, we selected several prospective reports from health visitors, parents and study participants to indicate harmful environments (ages 7, 11 and 16 years), including (i) impoverished upbringing [22, 23], (ii) hazardous conditions [24, 27], (iii) anti-social behaviours [28, 29] and (iv) poor parent-child relationships [30]. Indicators selected to represent these four child-to-adolescence domains are listed in Table 2. For example, impoverished upbringing was represented by family contact with various welfare agencies (e.g. social services). Hazardous conditions were represented by hospital admissions and occurrence of injuries (e.g. fractures and burns). Anti-social behaviours were represented by indicators of dysfunction or distress (e.g. contact with probation officers). Poor parent-child relationships were represented by the participant’s response at 16 years on how well s/he got on with their parent(s). The latter is notable in being the only self-assessment by participants. Most individual items within the domains of harmful environments were uncommon, e.g. prevalence of family contact with each identified welfare agency was < 4%, suggesting severe circumstances for affected individuals. Participants were classified at 7 years, 11 years and 16 years as having impoverished upbringing, hazardous conditions or antisocial behaviours if they had ≥ 1 item; parent-child relationships were represented only at 16 years (Table 2).

**Table.2:** Prospectively recorded information on harmful environments in childhood and adolescence in the 1958 British birth cohort

	Ascertainment age (reporter)^1^	1958 cohort variable	*N* (%)
**Impoverished upbringing**			
Family contact with any of:	7 years (HV)	Dr. Barnardo’s/other Children’s society	24 (0.32)
	7 years (HV)	Children’s department	224 (2.99)
	7 years (HV)	National Society/RSS^2^ for prevention of cruelty to children	68 (0.92)
	7 years (HV)	Psychiatric social worker	86 (1.17)
	7 years (HV)	Probation officer	105 (1.43)
	7 years impoverished upbringing^3^		393 (5.16)
	11 years (P)	Children’s department	145 (1.67)
	11 years (P)	Health department	78 (0.90)
	11 years (P)	Voluntary services	173 (1.99)
	11 years (P)	Criminal services	125 (1.44)
	11 years (P)	In care of local authority/voluntary society	220 (2.78)
	11 years impoverished upbringing^3^		573 (6.60)
	16 years (P)	Social services	313 (3.69)
	16 years (P)	Police probation	254 (2.99)
	16 years (P)	Voluntary services	52 (0.61)
	16 years impoverished upbringing^3^		519 (6.11)
	**7–16 years**^**4**^	**Impoverished upbringing**	**1166 (12.6)**
**Hazardous conditions**			
	7 years (P)	Concussion/head injury (with unconsciousness)	250 (3.07)
	7 years (P)	Hospital admission due to home (e.g. burns, scalds,poisoning), traffic or other accident^5^	110 (1.36)
	7 years hazardous conditions^3^		340 (4.16)
	11 years (P)	3+ hospital admissions	361(4.56)
	11 years (P)	2+ injuries (unconscious, fractures, wounds, burns)	274 (3.47)
	11 years hazardous conditions^3^		607 (7.58)
	16 years (P)	3+ hospital admissions due to accident^6^	414 (4.73)
	**7–16 years**^**4**^	**Hazardous conditions**^7^	**912 (10.0)**
**Participant’s anti-social behaviours**			
	7 years (HV)	Contact with school welfare/attendance officer	208 (2.89)
	7 years (P)	Destroys own/other’s belonging^8^	205 (2.52)
	7 years (P)	Disobedient at home^9^	283 (3.47)
	7 years (P)	Fights with other children^8^	412 (5.08)
	7 years anti-social behaviours^3^		894 (10.9)
	11 years (P)	Destroys other’s belongings^8^	69 (0.87)
	11 years (P)	Disobedient at home^9^	186 (2.33)
	11 years (P)	Fights with other children^8^	290 (3.68)
	11 years anti-social behaviours^3^		479 (6.00)
	16 years (P)	Contact with educational welfare	347 (4.09)
	16 years (P)	Contact with police probation	426 (5.02)
	16 years (P)	Destroys own/other’s property^8^	28 (0.40)
	16 years (P)	Disobedient^9^	130 (1.85)
	16 years (P)	Fights/extremely quarrelsome with other children^8^	97 (1.38)
	16 years anti-social behaviours^3^		846 (9.97)
	**7–16 years**^**4**^	**Anti-social behaviours**	**1830 (19.8)**
**Parent-child relationship**			
	16 years **(S)**	**Poor parent-child relationship**^**10**^	**757 (10.5)**
**Any harmful childhood environment**			
	**7–**16 years	0	5735 (61.9)
		1	2543 (27.5)
		2	839 (9.06)
		3/4	144 (1.55)

*N* varies from 7018 (16 years fights/extremely quarrelsome with other children) to 8747 (16 years 3+ hospital admissions due to accident) due to missing data

^1^Parent (P), health visitor (HV), S (self-report)

^2^Royal Scottish Society

^3^Within domain (impoverished upbringing, hazardous conditions or anti-social behaviours) classification of having 1+ item at 7 years, 11 years or 16 years

^4^Within domain problem at 7 years or 11 years or 16 years

^5^2+ different reasons why admitted to hospital

^6^If 16 years hospital data was missing, filled in with 11 years data

^7^Childhood hazardous conditions 7**–**16 years refers to participants with 7 years hazardous conditions or 2+ listed injuries by 11 years or 3+ hospital admissions by 16 years

^8^Frequently at 7 years/11 years; certainly applies at 16 years

^9^Frequently at 7 years/11 years; often at 16 years

^10^Answered very untrue or untrue to questions on getting on well with either their mother or father

#### Adult mental health

At 45 years, nurses administered the revised Clinical Interview Schedule [37], during which symptoms of depression and anxiety in the previous week were recorded. Participants reporting ≥ 2 items for the depressive symptoms or anxiety modules were identified as having elevated symptom levels (*n* = 1160; 12.5%). Our measure does not allow identification of clinical depression, but we refer henceforth to elevated symptoms as *depression*.

### Statistical analysis

In preliminary analyses, we first calculated simple percentages of prospectively recorded harmful environments in childhood, by adult retrospective report of abuse. Second, we used logistic regression to estimate odds ratios (*OR*s) and 95% confidence intervals (*CI*s) of prospective childhood harm by adult retrospective reports of abuse. Note that, as pertinent to our research questions, ORs are estimated for childhood harm ‘outcomes’ related to adult report of abuse in childhood. There was little evidence that associations differed by sex as tested with interactions (*p*_sex*any_abuse_ ≥ 0.19 for 7–16 years harmful environment domains); hence, analyses were sex adjusted. We also investigated associations separately for specific types of child abuse and harmful environments at each age in childhood. Third, we calculated positive predictive values (PPVs) [38] to estimate the chance that a participant who retrospectively reported abuse had a particular indicator for childhood harm. To assess the possibility of recall bias by current adult depression status, we examined (i) prevalence of retrospectively reported abuse and prospectively recorded harmful environments (7–16 years) by 45 years depression and (ii) PPVs stratified by depression status. Missing data ranged from 0.53% (45 years depression) to 22.8% (16 years parent-child relationship). Data loss was minimised by imputing missing data on 7 years, 11 years, and 16 years harmful childhood environments and 45 years depression using multiple imputation chained equations. Imputation models included all model variables and main predictors of missingness (i.e. childhood internalising and externalising behaviours, social class and cognition) [32]. Regression analyses were run across 20 imputed datasets and overall estimates obtained. Imputed results were similar to those using observed values; the former are presented.

## Results

Prevalence of retrospectively reported child abuse ranged from 10.7% (psychological) to 1.60% (sexual), with 14.8% recalling at least one type of abuse (Table 1). In general, prevalence of prospectively recorded harmful environments was similarly uncommon (Table 2), e.g. approximately 10% of participants reported poor parental relationships when they were 16 years and hazardous conditions 7–16 years, whilst 12.6% had an impoverished upbringing 7–16 years.

Adults who reported child abuse were more likely to have had at least one prospective indicator of harmful environment (52.4%) compared with 35.6% among the non-abuse group (Table 3). All types of retrospectively reported abuse differed from non-abuse in showing greater prevalence for each indicator of harmful environment at 7 years, 11 years and 16 years, except for hazardous conditions (Additional file 1: Tables S1 and S2). Accordingly, adult report of any type of abuse was associated with higher odds of prospective indicators of harm, including impoverished upbringing, anti-social behaviours and poor parent-child relationships at all childhood ages (Table 4 and Additional file 1: Table S2). In particular, all types of retrospectively reported abuse were associated with approximately three-fold higher odds of poor relationships with parents during adolescence (16 years); for any type of reported abuse, the *OR*_sex-adjusted_ was 2.98 (2.50, 3.54). Although *CI*s were wide, associations for retrospectively reported sexual abuse were consistently large, e.g. *OR*_sex-adjusted_ for impoverished upbringing was 4.49 (3.14, 6.43) compared with 2.02 (1.71, 2.40) to 2.86 (2.35, 3.50) for other types of abuse. A dose-response relationship was observed between retrospectively reported abuse and number of prospective indicators of harm with the relative risk ratio increasing from the reference group (no harms) to 1.75 (1.50, 2.03) for 1 harm and to 4.68 (3.39, 6.45) for 3 or 4 harms. Again, the trend for sexual abuse was striking, increasing from the reference group to 1.93 (1.19, 3.13) for 1 harm and to 14.4 (7.94, 26.0) for 3 or 4 harms.

**Table.3:** Prevalence of prospectively recorded harmful environments in childhood and adolescence by retrospective reports of child abuse (observed data)

	Retrospective reports of child abuse									
Prospective measures	Any abuse^**1**^		Physical abuse		Psychological abuse		Witnessing abuse		Sexual abuse	
	Yes (%)	No (%)	Yes (%)	No (%)	Yes (%)	No (%)	Yes (%)	No (%)	Yes (%)	No (%)
Impoverished upbringing (7**–**16 years)	21.5	11.1	23.8	11.9	21.1	11.6	27.5	11.7	37.8	12.2
Hazardous conditions (7**–**16 years)	10.5	9.91	11.6	9.90	10.1	9.99	9.74	10.0	8.97	10.0
Anti-social behaviours (7**–**16 years)	27.0	18.6	30.0	19.2	25.9	19.1	29.7	19.2	32.7	19.6
Poor parent-child relationships (16 years)	22.5	8.52	26.6	9.52	23.8	8.99	25.7	9.63	31.8	10.2
Any harmful environment (7**–**16 years)										
0	47.6	64.4	41.6	63.2	48.0	63.6	42.3	63.2	39.2	62.3
1	32.7	26.6	35.9	26.9	32.6	26.8	34.4	27.0	28.4	27.4
2	16.4	7.78	18.2	8.47	16.1	8.21	19.2	8.41	23.7	8.82
3/4	3.35	1.24	4.29	1.38	3.32	1.34	4.14	1.39	8.78	1.44

*N* varies due to missing data

Details given in Table 2

^1^At least one of physical, psychological, witnessing or sexual abuse (see Table 1 for definitions)

**Table.4:** Odds ratios (*95% CI*) of harmful environments in childhood and adolescence by retrospective reports of child abuse (imputed data, *N* = 9308)

	Retrospective reports of child abuse				
Prospective measures	Any abuse	Physical abuse	Psychological abuse	Witnessing abuse	Sexual abuse
Impoverished upbringing (7**–**16 years)	2.19 (1.89, 2.54)	2.31 (1.87, 2.85)	2.02 (1.71, 2.40)	2.86 (2.35, 3.50)	4.49 (3.14, 6.43)
Hazardous conditions (7**–**16 years)	1.15 (0.95, 1.39)	1.22 (0.93, 1.61)	1.09 (0.87, 1.36)	1.07 (0.80, 1.43)	1.25 (0.71, 2.20)
Anti-social behaviours (7**–**16 years)	1.73 (1.50, 1.99)	1.86 (1.53, 2.27)	1.59 (1.35, 1.88)	2.01 (1.64, 2.45)	2.45 (1.70, 3.55)
Poor parent-child relationships (16 years)	2.98 (2.50, 3.54)	3.49 (2.80, 4.34)	3.06 (2.52, 3.71)	3.12 (2.46, 3.95)	3.66 (2.42, 5.54)
Any harmful environment^~^ (7**–**16 years)					
0	Ref	Ref	Ref	Ref	Ref
1	1.75 (1.50, 2.03)	2.08 (1.67, 2.59)	1.69 (1.42, 2.02)	2.05 (1.64, 2.55)	1.93 (1.19, 3.13)
2	3.02 (2.50, 3.64)	3.49 (2.68, 4.56)	2.77 (2.23, 3.45)	3.90 (3.02, 5.03)	4.78 (2.97, 7.69)
3/4	4.68 (3.39, 6.45)	5.79 (3.80, 8.82)	4.29 (2.95, 6.24)	5.94 (3.87, 9.12)	14.4 (7.94, 26.0)

Sex-adjusted

~Relative risk ratios (from multinomial logistic regression) for any harmful environment (7–16 years)

As expected, the prevalence of retrospectively reported abuse and prospective indicators of harm was generally higher among participants identified as depressed than the non-depressed (Additional file 1: Table S3). However, for retrospectively reported abuse, the PPV of at least 1 prospective indicator of harm was similar for the depressed (0.58 (0.52, 0.64)) and non-depressed (0.58 (0.55, 0.61)), i.e. both groups were comparable to the population PPV (Table 5). Likewise, PPV for depressed and non-depressed groups were similar for each indicator of harm, i.e. impoverished upbringing, hazardous conditions, anti-social behaviours and poor parent-child relationships, e.g. PPV for poor relationships with parents was the same for depressed (0.23(0.17, 0.28)) and non-depressed (0.23(0.20, 0.25)) groups.

**Table.5:** Positive predictive value for (i) total population and (ii) stratified by 45 years depression

	Total	Depressed	Non-depressed
Any harmful environment (0 vs. 1+)	0.58 (0.55, 0.61)	0.58 (0.52, 0.64)	0.58 (0.55, 0.61)
Impoverished upbringing (7–16 years)	0.23 (0.21, 0.26)	0.24 (0.19, 0.29)	0.23 (0.21, 0.26)
Hazardous conditions (7–16 years)	0.12 (0.10, 0.14)	0.09 (0.06, 0.13)	0.12 (0.10, 0.15)
Anti-social behaviours (7–16 years)	0.30 (0.27, 0.33)	0.34 (0.28, 0.39)	0.29 (0.26, 0.32)
Poor parent-child relationships (16 years)	0.23 (0.20, 0.25)	0.23 (0.17, 0.28)	0.23 (0.20, 0.25)

Positive predictive value: probability of having at least one prospectively recorded harmful environment (7**–**16 years) among those who retrospectively reported child abuse at 45 years

## Discussion

In a large general population, we investigated whether adult retrospective reports of child abuse were associated with prospectively measured harmful environments in childhood and potential bias in retrospectively reported abuse by adult depression. We found that a majority of adults retrospectively reporting abuse had evidence of harm in childhood (52.4% versus 35.6% for others). Of particular note was the strong association, namely three-fold higher odds, between retrospectively reported abuse and poor relationship with parent(s) recorded when participants were adolescents. Interestingly, the relationship between retrospectively reported abuse and number of prospective indicators of harmful environments showed a dose-response trend with increasing relative risk ratios from no harms (reference group) to 4.68 (3.39, 6.45) for 3 or 4 harms. With regard to potential recall bias, for retrospectively reported child abuse, the probability of prospectively recorded harmful environments was similar among depressed and non-depressed adults. Thus, our study supports two main conclusions: firstly, our findings suggest that adult report of child abuse is related to environments in childhood that have been linked to abuse; secondly, our findings suggest that among those reporting child abuse, there was negligible impact of adult depression status on retrospective report of child abuse.

Our study has several strengths, largely relating to its use of data from a population-based birth cohort followed over several decades of life. In particular, a major advantage is the simultaneous availability of information on adult retrospective report of child abuse as well as relevant, wide-ranging prospective indicators of harmful environments in childhood, from parents, health visitors and, importantly, the participant themselves. Many individual items selected to indicate harm identified uncommon events or experiences, suggesting they may reflect severe circumstances. Other strengths include availability of information on depression status at the same time as child abuse reports, allowing investigation of possible recall bias. Questions about abuse were drawn from known instruments [33] and were completed confidentially by participants using a portable computer. Whilst more recent research quantifies child maltreatment using instruments that ask about experiences of behaviourally specific acts/events, as in the Juvenile Victimisation Questionnaire [39], our study asks respondents to identify their experiences as abuse. Moreover, our questions relate specifically to abuse by parents and frequency and duration are unknown. Inevitably, such differences in child abuse questions will affect prevalence estimates, although reassuringly, estimates in our study are similar to other UK studies based on the Juvenile Victimisation Questionnaire [40]. However, for child sexual abuse, our estimate is low, possibly due to the observation that perpetrators are less likely to be parents [41]. Given that we lacked prospective abuse data, we were unable to compare prospective versus retrospective reports of child abuse. Instead, our study was devised to explore the utility of adult reports of child abuse in order to investigate their appropriateness for research purposes, for example, to shed light on whether retrospective reports reflected childhood harms that might provide confidence (or otherwise) for studies of long-term health effects associated with adult report of child abuse. In that adult report was found to be associated with various indicators of harm, it could be argued that our study supports the use of such indicators of harm as predictors of childhood abuse in individuals. However, we caution against this and emphasise that the prospective indicators of harmful environments used here should not be taken as predictors for physical, sexual or psychological abuse (even though there might be an increased risk) in individuals. In respect of our purpose here, our study was designed to identify whether adults who retrospectively reported abuse were exposed to harmful childhood environments assessed prospectively, but it is unable to identify adults not reporting abuse who nonetheless experienced it. We only considered current depression as a potential influence on recall bias. Although this is regarded as a main putative influence on retrospective reports, other factors such as problems in long-term and working memory [42] could influence retrospective report. Data on prospective harms were collected from multiple sources (i.e. informants) and the general consistency of findings across the range of harmful environments is reassuring, although in some instances, the informant (i.e. parent) will be the abuse perpetrator. With over 30 individual prospective items of information over three ages to characterise harmful environments from childhood to adolescence, our measures are extensive but may nevertheless miss key dimensions of harm. Selection of prospective indicators was limited by available evidence: we acknowledge this inherent limitation within the context of the widely accepted view that there is no gold standard method of identifying or predicting abuse with certainty [16]. Given potential mismatches in the constructs of child and adulthood data (in common with other studies [18]) and without information for adult non-abuse reports among individuals experiencing child abuse, we can only partially address the issue of whether adults accurately report childhood experiences. Participants were 45 years when they recalled abuse and not all study participants survived to that age (6.7% died). However, over half of the deaths occurred before 7 years, mostly in the first few months of life [32]. Despite loss to follow-up participants at 45 years were broadly representative of the original population, although inevitably, the most disadvantaged were least likely to remain [32]. Hence, the possibility of selection bias must be acknowledged. Finally, to avoid further reduction in the sample due to missing information, we used multiple imputation.

Our study provides insights into the meaning of retrospectively reported child abuse in relation to prospectively recorded harmful environments in childhood; thereby it contributes to ongoing debates about retrospective and prospective reports of abuse [15, 18, 43, 44]. Importantly, our demonstration that adult retrospective report of abuse is associated with prospectively measured harm is not necessarily at odds with studies showing poor agreement between prospective and retrospective reports of child abuse [18], because measurement misclassification (e.g. due to completeness of case capture or differences in constructs) at either timepoint would weaken levels of agreement. Our observation that 52% of adults retrospectively reporting abuse had prospectively recorded measures of harm is consistent with another study showing 66% of adults recalling either abuse or neglect had prospective evidence of court substantiated maltreatment in childhood [45]. This consistency between our study and previous work is noteworthy, given that measures of childhood harm are from different sources (respectively, reports by parents, health visitors and participants vs. court-substantiated maltreatment). Prospective evidence of harm was lacking for a substantial proportion, almost half of the adult report child abuse group in our study. This finding is unsurprising because, whilst false memory and recall biases remain possibilities, more plausibly we have only partially detected childhood harm given that much abuse remains hidden and the inherent difficulties mentioned above regarding prediction/identification with proxy variables.

Our finding that adult retrospectively reported abuse is strongly associated with prospectively measured poor relationship with parents is particularly important as the latter was reported by participants themselves during adolescence, and hence, it is likely to be a key prospective measure in this population. The three-fold higher odds of poor parent-child relationships observed for the abuse reporting group is consistent with a meta-analysis showing strong relationships between physical abuse and high family conflict and low family cohesion [30]. All specific forms of abuse were associated with harmful environments in childhood and adolescence. This finding is consistent with the interpretation that, irrespective of the type of abuse examined here, adults who report a history of childhood abuse were likely to have lived in difficult circumstances as children. Yet, despite this supportive evidence, our study does not entirely resolve the issue of whether retrospective reports are valid for all different types of abuse. Caution in interpreting our results on type of abuse is needed because of the tendency for different types of abuse and other childhood adversities to co-occur, as described as multi-type maltreatment and polyvictimisation [46]. Such co-occurrence of childhood adversities needs to be considered when investigating whether effects vary for different types of child maltreatment on long-term health outcomes. Our finding of associations for all types of adult report of child abuse with prospective indicators of harm is not incompatible with the notion of varying long-term effects on outcomes. Indeed, varying associations with later adult outcomes have been demonstrated in our study population [21] taking account of multi-type maltreatment and polyvictimisation. Notwithstanding study shortcomings on validity for types of abuse, it is notable that associations for sexual abuse were consistently large: adult retrospective report was associated with a 14-fold risk of 3 or 4 harmful childhood environments compared with an estimated five-fold risk for retrospective report of any form of abuse. This finding suggests that adult retrospective report of sexual abuse by a parent, a rare occurrence (1.6%) in our population, is associated with a heavier burden of prospectively assessed childhood harm. Our finding for adult retrospective report is particularly relevant given that prospective measures such as court substantiated sexual abuse have high rates of false negatives [45]. More generally, with retrospectively reported abuse having an estimated PPV of prospective harm of about 0.6, our study provides further support for previous observations that when abuse or neglect is retrospectively reported to have taken place, these positive reports are likely to be correct [47].

An acknowledged issue regarding retrospective reports of child abuse is the potential for recall bias, as life experiences following abuse may alter an individual’s memory of the abuse. For example, depression has been shown to influence the reporting of traumatic childhood experiences [13]. The potential for recall bias in the 1958 cohort study is underscored by associations of adult reports of child abuse with poorer lifetime mental health trajectories from child to adulthood [48] and by observations mentioned here (concurrently at the time of adult reporting, 28% of depressed vs. 13% of non-depressed reported abuse). However, given the well-established associations of child abuse with poor later outcomes, including mental health [1, 48], differences in prevalence could have explanations other than recall bias. Our findings suggest that there was little recall bias in the reporting of child abuse because the probability of a harmful childhood environment (prospectively recorded) among the abuse group (retrospectively recorded) was the same for depressed and non-depressed groups at the time of abuse reporting. As expected, given that our study suggests that retrospective report of abuse was associated with harmful environments in childhood, we found that the prevalence of childhood harms was greater for the depressed than non-depressed group.

Whilst harmful childhood environments are not deterministic of abuse, the associations shown here to some extent corroborate adult reports of such events. This observation adds to evidence provided by our previous work showing these abuse measures to be associated with differing health and socio-economic life-courses, several years before and also after the timing of the adult abuse reports [6, 8, 21, 48–51]. Yet, problems associated with retrospective reports of child abuse remain unresolved to the extent that their validity has been called into question. In this context, our demonstration that adult report of child abuse is strongly linked to childhood harm assessed prospectively is an important contribution, providing some support for retrospective reports based research on child abuse. Thus, our study supports conclusions of others that unmitigated rejection of adult retrospective reports of abuse is unwarranted [47]. However, a challenge going forward is to improve the accuracy of child abuse measures, e.g. by use of multi-method strategies using both self-report and child protection service records [14] and, ideally, with further refinements to self-report instruments. Improvements to measurement are needed because general populations, such as the 1958 cohort, are crucial for understanding the long-term impact of child abuse on adult health, intervening pathways and moderating factors. Such understanding in turn informs the development of potential interventions to prevent or reduce the extent of negative outcomes of abuse in childhood.

## Conclusions

In summary in a large, unselected population cohort, we demonstrate that adult retrospective report of childhood abuse is linked, with a dose-response pattern, to prospective measures of harmful childhood environments. Importantly, we found strong associations between adult reports and poor relationships with parent(s), as self-reported by participants during adolescence. Finally, we found little evidence of recall bias for retrospectively reported child abuse in the 1958 birth cohort by adult depression status. Our study therefore strengthens the validity of research that relies on adult retrospective reports of childhood abuse.

## Supplementary Information

**Additional file 1: Tables S1-3.** additional supplementary data.

## Data Availability

All data can be accessed via the UK Data Service.

## Abbreviations

CI: Confidence interval
OR: Odds ratio
PPV: Positive predictive value
