# Supplementary material for: Adult retrospective report of child abuse and prospective indicators of childhood harm: a population birth cohort study
Source: BMC Med. 2021 Nov 29;19:286. doi: 10.1186/s12916-021-02164-5 (PMC8628375; doi:10.1186/s12916-021-02164-5)
Supplement: Supplementary file 1 — Additional file 1: Tables S1-3. additional supplementary data. [file 12916_2021_2164_MOESM1_ESM.docx]

Table S1: Prevalence of prospectively recorded harmful environments in childhood and adolescence by retrospectively reported child abuse (observed data)

|  | **Retrospectively reported child abuse** | | | | | | | | | | | | | |
| --- | --- | --- | --- | --- | --- | --- | --- | --- | --- | --- | --- | --- | --- | --- |
| **Prospective measures** | **Any abuse** | | **Physical abuse** | | **Psychological abuse** | | **Witnessing abuse** | | **Sexual abuse** | | | | | |
|  | Yes (%) | No (%) | Yes (%) | No (%) | Yes (%) | No (%) | Yes (%) | No (%) | Yes (%) | No (%) | | | | |
| **Impoverished upbringing** | | | | | | | | | | | | | | |
| 7y | 10.1 | 4.32 | 12.3 | 4.71 | 10.1 | 4.58 | 11.0 | 4.80 | 23.9 | | 4.88 | | |  |
| 11y | 12.1 | 5.66 | 12.1 | 6.25 | 12.5 | 5.89 | 13.4 | 6.17 | 18.1 | | 6.42 | | |  |
| 16y | 10.3 | 5.39 | 11.6 | 5.77 | 10.1 | 5.64 | 16**.0** | 5.50 | 22.4 | | 5,85 | | |  |
| 7-16y | 21.5 | 11.1 | 23.8 | 11.9 | 21.1 | 11.6 | 27.5 | 11.7 | 37.8 | | 12.2 | | |  |
| **Hazardous conditions** | | | | | | | | | | | | |  |  |
| 7y | 4.17 | 4.16 | 4.23 | 4.16 | 3.54 | 4.23 | 3.59 | 4.20 | 4.17 | | 4.16 | | |  |
| 11y | 8.52 | 7.43 | 10.4 | 7.41 | 8.59 | 7.47 | 8.37 | 7.54 | 6.36 | | 7.60 | | |  |
| 16y | 5.23 | 4.65 | 5.14 | 4.71 | 5.37 | 4.66 | 5.42 | 4.69 | 5.97 | | 4.71 | | |  |
| 7-16y | 10.5 | 9.91 | 11.6 | 9.90 | 10.1 | 9.99 | 9.74 | 10.0 | 8.97 | | 10.0 | | |  |
| **Anti-social behaviours** | | | | | | | | | | | |  |  |  |
| 7y | 15.3 | 10.2 | 18.1 | 10.5 | 15.2 | 10.4 | 15.8 | 10.6 | 20.7 | | 10.8 | | |  |
| 11y | 8.19 | 5.63 | 10.2 | 5.74 | 7.54 | 5.82 | 8.39 | 5.85 | 7.27 | | 5.98 | | |  |
| 16y | 15.0 | 9.10 | 16.1 | 9.58 | 14.7 | 9.40 | 18.8 | 9.42 | 20.9 | | 9.79 | | |  |
| 7-16y | 27.0 | 18.6 | 30.0 | 19.2 | 25.9 | 19.1 | 29.7 | 19.2 | 32.7 | | 19.6 | | |  |
| **Poor parent-child relationships** |  | |  |  |  |  |  |  |  | |  | | |  |
| 16y | 22.5 | 8.52 | 26.6 | 9.52 | 23.8 | 8.99 | 25.7 | 9.63 | 31.8 | | 10.2 | | |  |
| **Any harmful environment** 7-16y 0 | 47.6 | 64.4 | 41.6 | 63.2 | 48.0 | 63.6 | 42.3 | 63.2 | 39.2 | | 62.3 | | |  |
| 1 | 32.7 | 26.6 | 35.9 | 26.9 | 32.6 | 26.8 | 34.4 | 27.0 | 28.4 | | 27.4 | | |  |
| 2 | 16.4 | 7.78 | 18.2 | 8.47 | 16.1 | 8.21 | 19.2 | 8.41 | 23.7 | | 8.82 | | |  |
| 3/4 | 3.35 | 1.24 | 4.29 | 1.38 | 3.32 | 1.34 | 4.14 | 1.39 | 8.78 | | 1.44 | | |  |

N varies due to missing data

Table S2: Odds ratios* (95% CI) of harmful environments in childhood and adolescence by retrospectively reported child abuse (imputed data, N=9308)

|  | **Retrospectively reported child abuse** | | | | |
| --- | --- | --- | --- | --- | --- |
| **Prospective measures** | **Any abuse** | **Physical abuse** | **Psychological abuse** | **Witnessing abuse** | **Sexual abuse** |
| **Impoverished upbringing** | | | | | |
| 7y | 2.40 (1.91, 3.01) | 2.72 (2.02, 3.68) | 2.30 (1.78, 2.95) | 2.45 (1.82, 3.30) | 5.97 (3.87, 9.19) |
| 11y | 2.27 (1.87, 2.76) | 2.07 (1.60, 2.73) | 2.27 (1.83, 2.82) | 2.36 (1.81, 3.07) | 3.22 (2.04, 5.07) |
| 16y | 2.02 (1.62, 2.50) | 2.12 (1.59, 2.84) | 1.87 (1.46, 2.40) | 3.24 (2.50, 4.20) | 4.65 (3.07, 7.03) |
| 7-16y | 2.19 (1.89, 2.54) | 2.31 (1.87, 2.85) | 2.02 (1.71, 2.40) | 2.86 (2.35, 3.50) | 4.49 (3.14, 6.43) |
| **Hazardous conditions** | | | | | |
| 7y | 1.05 (0.76, 1.45) | 1.04 (0.65 ,1.66) | 0.90 (0.60, 1.35) | 0.89 (0.53, 1.48) | 1.17 (0.48, 2.86) |
| 11y | 1.19 (0.96, 1.48) | 1.36 (1.01,1.83) | 1.18 (0.92, 1.52) | 1.19 (0.86, 1.66) | 1.29 (0.67, 2.49) |
| 16y | 1.20 (0.91, 1.57) | 1.07 (0.72, 1.60) | 1.23 (0.90, 1.67) | 1.27 (0.86, 1.90) | 1.68 (0.82, 3.47) |
| 7-16y | 1.15 (0.95, 1.39) | 1.22 (0.93, 1.61) | 1.09 (0.87, 1.36) | 1.07 (0.80, 1.43) | 1.25 (0.71, 2.20) |
| **Anti-social behaviours** | | | | | |
| 7y | 1.65 (1.38, 1.97) | 1.85 (1.44, 2.36) | 1.62 (1.31, 1.99) | 1.67 (1.29, 2.17) | 2.52 (1.62, 3.93) |
| 11y | 1.57 (1.24, 1.99) | 1.92 (1.40, 2.63) | 1.38 (1.05, 1.82) | 1.60 (1.14, 2.24) | 1.55 (0.70, 3.42) |
| 16y | 1.85 (1.55, 2.20) | 1.85 (1.45, 2.36) | 1.74 (1.43, 2.14) | 2.36 (1.86, 3.02) | 2.96 (1.95, 4.51) |
| 7-16y | 1.73 (1.50, 1.99) | 1.86 (1.53, 2.27) | 1.59 (1.35, 1.88) | 2.01 (1.64, 2.45) | 2.45 (1.70, 3.55) |
| **Poor parent-child relationships** | | | | | |
| 16y | 2.98 (2.50, 3.54) | 3.49 (2.80, 4.34) | 3.06 (2.52, 3.71) | 3.12 (2.46, 3.95) | 3.66 (2.42, 5.54) |
| **Any harmful environment**^~^ **(**7-16y) | | | | | |
| 0 | Ref | Ref | Ref | Ref | Ref |
| 1 | 1.75 (1.50,2.03) | 2.08 (1.67,2.59) | 1.69 (1.42,2.02) | 2.05 (1.64,2.55) | 1.93 (1.19,3.13) |
| 2 | 3.02 (2.50,3.64) | 3.49 (2.68,4.56) | 2.77 (2.23,3.45) | 3.90 (3.02,5.03) | 4.78 (2.97,7.69) |
| 3/4 | 4.68 (3.39,6.45) | 5.79 (3.80,8.82) | 4.29 (2.95,6.24) | 5.94 (3.87,9.12) | 14.4 (7.94,26.0) |

*sex adjusted; ^~^Relative risk ratios (from multinomial logistic regression) for any harmful environment (7-16y)

Table S3: Prevalence of retrospectively reported child abuse and prospectively recorded harmful environments (7-16y) by 45y depression (observed data)

|  | Depressed  (N=1160) | Non-depressed  (N=8099) |
| --- | --- | --- |
| *Retrospectively reported (45y) child abuse* | | |
| Any abuse | 323 (27.8) | 1054 (13.0) |
| Physical abuse | 147 (12.7) | 414 (5.11) |
| Psychological abuse | 244 (21.0) | 754 (9.31) |
| Witnessing abuse | 121 (10.4) | 434 (5.36) |
| Sexual abuse | 45 (3.88) | 104 (1.28) |
| *Harmful childhood environments (7-16y)* | | |
| Impoverished upbringing | 187 (16.2) | 971 (12.1) |
| Hazardous conditions | 103 (9.07) | 808 (10.2) |
| Anti-social behaviours | 304 (26.3) | 1522 (19.0) |
| Poor parent-child relationships | 122 (14.0) | 635 (10.1) |
| Any harmful environment 0 | 644 (55.6) | 5054 (62.7) |
| 1 | 337 (29.1) | 2197 (27.3) |
| 2 | 153 (13.2) | 684 (8.49) |
| 3/4 | 24 (2.07) | 120 (1.49) |

N varies due to missing data

p-value for associations with 45y depression from Pearson's chi-squared test for hazardous conditions=0.25; for all retrospectively reported child abuse and all other prospectively recorded harmful childhood environments ≤0.001
